# Supplementary material for: Complexome profiling of the Chlamydomonas psb28 mutant reveals TEF5 as an early PSII assembly factor
Source: Plant Cell. 2025 Mar 17;37(6):koaf055. doi: 10.1093/plcell/koaf055 (PMC12164590; doi:10.1093/plcell/koaf055)
Supplement: koaf055_Supplementary_Data [file koaf055_supplementary_data.zip › psb28_interactive.zip!/plots/intensity/qualityPlots/peptides/FUM1_Cre06.g254400.t1.1.html]

### Cre06.g254400.t1.1

Trivial name: FUM1  
SeqCoverage [median]: 10.70  
Mass: 63.49 kDa  
  
 

| Sequence | MissedCleavage | Mass [Da] | wtIntensity | muIntensity | Score |
| --- | --- | --- | --- | --- | --- |
| AYTSTNLR | 0 | 924.47 | 0.00 | 1073.96 | 73.62 |
| NPGQYLPDVTHEQLSTDVVK | 0 | 2239.11 | 0.00 | 3115.26 | 85.81 |
| TSLYQQTK | 0 | 967.50 | 393.91 | 0.00 | 74.13 |
| YLDTLPTSGNALGR | 0 | 1476.76 | 937.89 | 4622.44 | 120.09 |
| YSQVAPLDMFSEK | 0 | 1513.71 | 0.00 | 1358.88 | 79.69 |
|  |  |  | 1331.8 | 10170.5 |  |
